# Trust-building as a Keystone Activity in Beaver-related Restoration Practice

**DOI:** 10.1007/s00267-026-02400-9

**Published:** 2026-02-20

**Authors:** Brian D. Erickson, Megan S. Jones

**Affiliations:** 1 Department of Fisheries, Wildlife, and Conservation Sciences, Oregon State University, Corvallis, OR USA; 2 U.S. Geological Survey, Oregon Cooperative Fish and Wildlife Research Unit, Oregon State University, Corvallis, OR USA

**Keywords:** Beaver restoration, Wildlife coexistence, Trust-building, Trust repair, Qualitative research

## Abstract

North American beavers (*Castor canadensis*) are increasingly being used to achieve restoration goals, prompting practitioners to engage with private landowners in efforts to promote beaver coexistence. Through 23 semi-structured interviews with restoration practitioners in Oregon, USA, we explored how practitioners from government agencies, non-governmental organizations (NGOs), service organizations, and private businesses communicate with private landowners about nonlethal beaver management and habitat creation. Using abductive analysis, we identified trust-building as an essential element of restoration practice. Practitioners described 60 tactics for building trust, which we organized using the Shared Foundations model of trust and distrust and the adaptive management cycle to bridge theory with field-based experience. Practitioners also reported navigating tensions between tactics and adapting their approaches to individual landowners and contexts. We argue that trust-building is a craft that can be mastered, propose a potential progression from novice to master trust-builder, and highlight the need for greater attention to trust, relationships, and trust repair in environmental management. Our findings offer a theoretically grounded yet practitioner-informed framework for understanding and improving trust-building efforts in restoration practice.

## Introduction

Environmental managers in the American West face a myriad of well-known climate-related challenges, including species decline (Weiskopf et al. 2020), larger and more frequent wildfires (Higuera and Abatzoglou 2021), and more severe and widespread drought (Zhuang et al. 2024). Increasingly, North American beavers (*Castor canadensis*), as ecosystem engineers (Brazier et al. 2021; Lawton & Jones, 1995), are included as one of many climate mitigation solutions (Welden 2023). Beavers’ dam-building activities have the potential to increase wildfire resilience (Fairfax and Whittle 2020) as well as improve water quality and quantity, modify habitat in ways that benefit other species, and repair damaged riparian processes (Arkle and Pilliod 2015; ODFW, 2016, 2021).

However, beaver populations are less robust than they once were. Beavers were nearly extirpated by the 19th-century fur trade (ODFW 2025). Today, beaver populations are recovering (Kearns and West 2022; ODFW, 2025; Taylor 2021), but to realize the climate benefits of beavers, more beavers are needed on the landscape. In places like Oregon, USA, nearly 60% of land is publicly owned and beavers already occupy much of the available suitable habitat on public lands (ODFW 2021). Elsewhere, such as the eastern United States and Europe, private land makes up the majority of land ownership. In both instances, restoration practitioners are increasingly turning to conservation on private lands to increase beaver populations and obtain beaver-related benefits.

Encouraging beaver coexistence on private lands can present major communication and implementation challenges for restoration practitioners. On the one hand, beavers can provide placed-based benefits to certain private landowners, particularly in arid landscapes where they can support management goals by improving water storage for livestock, mitigating wildfire damage, and aiding with post-wildfire recovery (Welden 2023). However, although private landowners may care about environmental goals, beaver activities can be in tension with their other land management priorities by causing flooding and damaging property and crops (Charnley et al. 2020; Guziejka 2025). On working lands, which are areas actively managed for production such as farmland, orchards, ranches, forestland, nurseries, vineyards, and other agricultural operations purposes (Kremen and Merenlender 2018; Weathersby and Julian 2025), beavers may hold reputations as “nuisances” (Charnley et al. 2020; Enck et al. 1992; Fountain, 2014) and “pests” (Fountain 2014) that should be controlled or removed. In many locations, lethal removal of beavers is standard management practice (Fountain 2014; Horry County 2025; Jonker et al. 2009; Nash et al. 2021), although it may be less acceptable in Oregon (Morzillo and Needham 2015). Lethal beaver management practices can come at a cost to landowners, because trapping often costs more than coexistence (Hood et al. 2018), and to ecosystems, through impaired ecosystem services (Brazier et al. 2021; Burgher et al. 2026). Additionally, because of historical beaver removal and despite their foundational role in creating the landscapes that early settlers occupied (Nash et al. 2021), beavers are sometimes seen as unnatural new arrivals that do not belong in working landscapes. In summary, although beavers have the potential to help address multiple environmental challenges, convincing private landowners not to kill beavers, much less create habitat to attract them, can be difficult.

Nevertheless, restoration professionals and environmental managers have pursued beaver-related restoration on private lands for more than two decades (Burgher et al. 2026; Charnley, 2019; Charnley et al. 2020; Gottschalk Druschke et al. 2024; McKinstry & Anderson, 1999; Nash et al. 2021; Pilliod et al. 2018). Beaver-related restoration involves three techniques (Burgher et al. 2026): beaver mimicry (instream structures such as beaver dam analogs and low-rise rock dams), habitat restoration to attract beavers (planting beaver-preferred foods and creating conditions where beaver thrive), and beaver reintroduction (Auster et al. 2021; Fountain, 2014). Additionally, practitioners promote nonlethal damage mitigation (sand paint, plant protection, exclusion and flow devices, intentional plant choices, dam notching and removal, and relocation) (Kinas et al. 2024; Pollock et al. 2023) to keep beavers on the land and accrue ecosystem-wide benefits. Practitioners have attempted to gain practical experience and insight on what communication approaches help them effectively promote beaver-related restoration and human-beaver coexistence on private lands.

The research presented here arose from a larger study (Erickson and Jones 2026), in which we asked the following research question: how are restoration practitioners communicating with private landowners about nonlethal beaver damage mitigation and habitat creation for beavers? Although it was not the focus of the interviews, trust and relationship building emerged as foundational elements of practitioners’ approach to promoting beaver coexistence. Here, we present restoration professionals’ tactics for building trust with private landowners as well as the tensions they face while doing so. In the process, we highlight what practitioners do to build trust, grounding typically abstract trust constructs in concrete practitioner actions.

### Theoretical Background

Trust has received extensive coverage within the environmental management literature (Cvetkovich and Winter 2003; Davenport et al. 2007; Emborg et al. 2020; Smith et al. 2013; Vaske et al. 2007). It carries multiple benefits, such as increasing support for environmental management actions (Stern and Coleman 2015), promoting collaboration (Davenport et al. 2007), and reducing management costs (Liljeblad and Borrie 2006). Trust is typically defined as a willingness or “intention to accept vulnerability based upon positive expectations of the intentions or behavior of another” (Rousseau et al. 1998, p. 395). However, when exploring trust dynamics with landowners who have assets at risk, it can help to frame trust as “choosing to risk making something you value vulnerable to another person’s actions” (Feltman 2021, p. 9). This alternative definition shifts the focus from passive mental states to active decision-making, makes explicit that valuable things are at stake, and avoids assuming positive expectations about outcomes. Distrust, conceptualized as the opposite of trust (Erickson and Biedenweg 2025; Stern and Coleman 2015), can be defined as the determination that “what is important to me is not safe with this person in this situation” (Feltman 2021, p. 11). Distrust is often framed as an obstacle to conservation and changes in management practice (Nie 2003; Stern, 2008), and perceived differences in value similarity between residents and agency personnel is a predictor of distrust in managing agencies (Cvetkovich and Winter 2003).

Scholars have identified dozens of antecedents of trust (McEvily and Tortoriello 2011). The Shared Foundations Model of trust and distrust (Erickson and Biedenweg 2025) provides a comprehensive framework for organizing these various factors into four overarching categories. The model argues that whether an individual trusts or distrusts another party in natural resource management contexts depends on antecedents within four interconnected dimensions: (1) the perceived **vulnerability** of the individual doing the trusting (the trustor), (2) the perceived **trustworthiness** of the other party (the trustee), (3) the **relationship** between the trustor and trustee, and (4) the **situation**. This framework acknowledges that trust emerges through dynamic interactions between parties within specific contexts (Korsgaard et al. 2015), making it especially useful in environmental management settings where landowners have direct interests at stake. Although trust involves reciprocal relationships where each party serves as both trustor and trustee, for clarity, we present the model from the landowners’ perspective with landowners as trustors and practitioners as trustees because our focus is on exploring how practitioners build trust with landowners.

**Vulnerability** includes a combination of an individual’s perceived ***threat*** (does this matter for things they care about?) and ***coping potential*** (can they do something about it?) (Lazarus 1985). Drawing from Protection Motivation Theory (Floyd et al. 2000; Rogers, 1975, 1983), the greater the threat or the lesser the coping potential, the more vulnerable the landowner will likely feel. Broadly speaking, voluntary, controllable, and familiar threats are more acceptable to people than imposed, uncontrollable, and unfamiliar ones (SAMHSA 2019).

**Perceived trustworthiness** of practitioners is based on two overarching assessments: their intentions and whether they have the capability to carry out their intentions (Fiske et al. 2007). This can be broken down into landowner assessments of practitioners’ capability, reliability, integrity, and benevolence, which we call the “trustworthiness C.R.I.B.” as a memorable way to describe the foundations of trustworthiness (Mayer et al. 1995; McEvily and Tortoriello 2011; PytlikZillig et al. 2016). ***Capability*** focuses on whether the practitioner has the knowledge and skills to carry out their intentions (Dietz and Den Hartog 2006; Mayer et al. 1995; PytlikZillig et al. 2016). ***Reliability*** centers on whether the practitioner keeps their promises and commitments (Dietz and Den Hartog 2006; Feltman 2021). ***Integrity*** involves moral and ethical behavior, such as honesty, sincerity, and fair and respectful treatment of others (Mayer et al. 1995; PytlikZillig et al. 2016). ***Benevolence*** revolves around whether practitioners care for landowners’ well-being alongside their own interests (PytlikZillig et al. 2016), which includes assessments of value similarity and shared identity (Cvetkovich and Winter 2003; Siegrist, 2000).

**Relationships** between landowners and practitioners also play an important role in shaping trust. Whether the landowner is familiar with, likes, and feels trusted by the practitioner can influence levels of trust (Baer and Colquitt 2018; Korsgaard 2018). These considerations can serve as heuristics, or mental shortcuts, where a simpler question (“do I like them?”) substitutes for a more difficult question (“should I trust them to do this action in this context?”) (Baer and Colquitt 2018; Tversky and Kahneman 1974). These relational factors can provide additional data for making assessments of practitioners’ trustworthiness and landowners’ vulnerability.

**Situational factors**, including procedural trust (Stern and Coleman 2015), can encourage trustworthy behavior, justify untrustworthy behavior, and change the landowner’s vulnerability (Erickson and Biedenweg 2025). These contextual considerations include institutional structures, decision-making procedures, rule enforcement, resource constraints (Erickson and Biedenweg 2025; Sitkin and Roth 1993; Stern and Coleman 2015), and in this case, beavers themselves.

## Methods

### Study Context

This case study focuses on beaver coexistence communication in Oregon, USA, the “beaver state” (ODFW, n.d.). Oregonians hold a wide range of political beliefs ranging from very liberal to very conservative (Oregon Values and Beliefs Center 2023). They live in cities with populations ranging from 3 to over 652,000 people (Population Research Center 2025) and hold varied beliefs about how private and public lands and the environment should be managed (Oregon Values and Beliefs Center 2023). Ecologically, Oregon contains diverse habitats across its eight ecoregions, including woodlands, temperate rainforests, coastal dunes, grasslands, steppe, shrublands, alpine meadows, springs, seeps, streams, rivers, wetlands, estuaries, working lands, and urban areas (ODFW 2016).

Beaver populations (*Castor sp.)* are native and expanding across North America, Europe, and Asia (Brazier et al. 2021; Halley et al. 2021; Tape et al. 2022), and this includes in Oregon. Similar to other states and territories in North America (Fairfax and Westbrook 2024), there is no official estimate of the total beaver population in Oregon (ODFW 2023); however, unofficial estimates suggest there are over 1 million beavers in the state (Taylor 2021). Beavers are found in all Oregon ecoregions (Cafferata et al. 2023; ODFW, 2016), and Oregon Department of Fish & Wildlife (ODFW) considers their populations to be “doing well” (ODFW, n.d.).

As beaver populations grow, there is increasing interest in beaver-related restoration in Europe (Brazier et al. 2021) and North America (Burgher et al. 2026; Goldfarb & Flores, 2018; Koenigsberg, 2018) including in the Pacific Northwest (Charnley et al. 2020; Gottschalk Druschke et al. 2024; Nash et al. 2021). In Oregon, beavers could help with several conservation goals, including maintaining and restoring floodplains and wetlands, reducing sedimentation, improving water quality, and increasing habitat for various fish and wildlife species (Gottschalk Druschke et al. 2024; ODFW 2016). As such, supporting and encouraging beaver dam-building activity is one of the actions identified for addressing altered floodplain function in the Oregon Conservation Strategy (ODFW 2016). In 2023, following multiple beaver-related focus groups (Beaver Management Work Group 2022), ODFW released a three-year action plan for beaver-modified landscapes which focused on furthering the protection and restoration of beaver habitat and beaver-modified habitat in Oregon (ODFW 2023).

In 2024, rules related to the lethal removal (“take”) of beavers in Oregon changed with the passage of HB 3464 (Oregon Legislative Assembly 2023). Previously, beavers were managed as both furbearers, animals whose fur has commercial value (ODFW 2026), and rodent “agricultural predators,” animals that are or may be destructive to agricultural crops, products, and activities (ODFW 2024a). Furtakers, people who recreationally trap fur bearing animals, were required to have a license and follow regulations, but beavers causing damage on private property were defined as predatory animals and rodents, meaning there was unlimited take of beavers on private land without a permit (Kentnesse 2023).

After passage of HB 3464, beavers are no longer classified as agricultural predators. Lethal removal of beavers is still allowed on private land with a permit from ODFW; however, there are exceptions for “imminent threats” to infrastructure or agricultural crops and for certain small forestland owners (Kentnesse 2023; ODFW 2025). With a lethal control permit, landowners can either hunt or trap the beaver themselves, hire a permitted Wildlife Control Officer, or allow someone with a furtaker license to trap the beaver in season. All beaver take must now be reported. Oregon’s furbearer regulations allow in-season harvest of beavers without quotas or limits (ODFW 2024a). Furtakers have education, licensing, reporting, and trap checking requirements as well as location restrictions. Trapping data from 2023 showed 1495 beavers were harvested by 115 furtakers across 32 of 36 Oregon counties (Broman and Wolfer 2024), above the three-year (2021-2023) furtaker harvest average of 1231 beavers per year (ODFW 2024b).

### Study Procedures

We used semi-structured interviews with individuals who communicated with private landowners about beaver coexistence. We conceptualize coexistence as including both nonlethal mitigation actions as well as actively restoring habitat for beavers but not including beaver mimicry unless it is done with the intent of attracting beavers to the area. Potential interviewees were identified using purposive sampling (Patton 2002) of public records from prior beaver-related working groups, conferences, management plans, beaver-related grant recipients, and email lists. Additional individuals were identified using chain referral (Patton 2002). These methods generated a list of 258 potential interviewees, 40 of whom we invited to interview. We prioritized contacting interviewees from terrestrial ecoregions (ODFW 2016) and sectors (local, state and federal government agencies; conservation and restoration non-governmental organizations (NGOs); service agencies such as soil and watershed conservation districts and watershed councils; and private businesses). We refer to these individuals of varied professional roles collectively as “practitioners” for simplicity’s sake. We stopped interviews when we had talked with multiple practitioners from each region and sector, and when interviews appeared to reveal little new information about practitioner communication approaches (Table 1). Of the 40 individuals we contacted, seven declined, eight did not respond, one referred a colleague, and one invitation to participate was undeliverable, resulting in 23 interviewees. The lead author recruited seven interviewees at a beaver-related conference in November 2023, including one interviewee whom he already knew. The author team also had informational scoping calls with two interviewees after receiving funding but before initiating data collection.

**Table 1:** Interviewees by sector and ecoregion

	Ecoregion								
Sector	Blue mts.^*a*^	Coast range	Columbia plateau	East Cascades	Klamath mts.^*a*^	Northern basin & range	West Cascades	Willamette Valley	Total^*b*^
Business	1	1	1	1	1	1	1	2	**9**
Government	2	4	2	1	2	1	2	4	**18**
NGO	4	2	0	3	1	0	1	2	**13**
Service	1	1	1	1	0	1	0	3	**8**
**Total**^*b*^	**8**	**8**	**4**	**6**	**4**	**3**	**4**	**11**	

^*a*^ Mts. = mountains. ^*b*^ Totals add up to more than the total number of interviewees (*n* = 23) because some interviewees worked in multiple sectors or ecoregions.

Interviews took place online via Zoom between November 2023 and March 2024. The lead author conducted all interviews following an interview guide, which asked questions about the practitioners’ approaches to communicating before, during, and after beaver damage and beaver-focused restoration (Online Resource 1). No questions in the interview guide directly asked about trust or relationship building. Prior to interviews, the lead author emailed participants a consent document and answered questions. Interviews were audio-recorded with consent and ranged in duration from 43 to 100 minutes in length (mean = 73 min). Following each interview, the interviewer recorded notes to capture his initial thoughts and reflections (Bernard 2006; Emerson et al. 2011).

Each interview was transcribed by a transcription service. The lead author was the sole coder for all analysis and used MaxQDA to manage the coding process. He used an abductive approach (Dubois and Gadde 2002; Thompson 2022), cycling inductive discovery and deductive application of existing theory. In the first round of coding, he applied Initial Coding (to identify analytic leads) and In Vivo coding (using the participants’ own words as codes) (Saldaña 2016) and identified trust-building as a potential direction for further analysis. In the second round of coding, he identified multiple ways that practitioners talked about building trust. The author team decided, through discussion, to use the Shared Foundations Model as a framework for organizing the larger number of trust-building approaches found in the transcripts. Later, an additional layer of categorization was needed to better show and communicate how trust was built over time, and the authors decided to use the general stages of the adaptive management cycle (Plan, Do, and Learn) to satisfy this need (Bennett et al. 2022). The trust-building tactics were organized across the stages of adaptive management into a table, resembling a codebook. This table assigned names and provided one or more exemplary quotes for each tactic (Online Resource 2).

We validated our respondent findings (i.e., member checking) (O’Brien et al. 2014) in two ways. First, we shared the findings with interviewees (*n* = 8) who were participating in a beaver coexistence community of practice that the authors were leading. Additionally, once a full manuscript was ready, all interviewees were contacted to provide input on the overall findings, suggest gaps, and approve quotations for use. This project was approved by the lead authors’ university institutional review board (HE-2023-550).

## Results

**Trust** and **relationship-building** emerged as critical components of practitioners’ approaches to communicating with private landowners about beaver coexistence. In the sections below, we focus on three key insights that emerged from interviews.

### Insight 1: Practitioners Knew Trust and Relationships were Important

During interviews, practitioners regularly brought up the importance of trust and relationships for their work with private landowners. After spontaneously mentioning trust multiple times, the first interviewee remarked, “I can’t say the word trust enough” (I01). Another explained, “if there is one thing that I will viciously protect, it’s trust with my landowners because it’s the only avenue that I get work done” (I15). Practitioners explained that pre-existing trust-based relationships drove projects in some areas (Table 2). They also described how involving landowners in the process and communicating repeatedly over timespans of months and years also built trust.

**Table 2:** Example quotes describing the importance of trust-building while promoting beaver coexistence and restoration

Trust scenario	Quote
Starting with trust	*“Trust is a big thing, though […] I don't know if I could say it's part of culture, but we have lots of Forest Service, [Bureau of Land Management], [Oregon Department of Forestry]. Those are stable job providers in our community, but there's also a concern. You don't want the government to tell you what you can and can't do on your property. So partly, I think, it comes down to trust. If we come to them, there's always like, ‘Well, what's the catch? What, you're going to do this project for nothing? You are going to spend $500,000 on our property and we don't have to do anything?' [...] I think there's that point where they come to us, the trust is always constantly building, but they must already have a sense of trust. You don't have to start off with just gaining trust. It's already there a little bit.” - I17*
Building trust through process	*“Project planning's really fun. That's where you get to be creative, so bring [the landowner] in. ‘You're part of the design team. Let's work through this.’ And definitely make sure you vetted everything before you modify somebody's landscape. Don't come up with some idea, write a grant and then show up and say, ‘we're going to do this.’ I've seen people do that, and it's a disaster […] It's disrespectful. Bring them along and make sure that whatever it is you're planning works, isn't scary, it makes sense for whatever their management is. […] I mean, if the relationship is a trust-based relationship, which they are, and then you're potentially taking risks that they're uncomfortable with, that's a pretty sketchy ground to be on. Ultimately, you want a long-term relationship with whoever it's doing restoration work with, and really you want them to have positive experiences with this type of work so that they're interested in doing it in the future. Even if your plan is to move on, you don't want to damage the credibility of doing that kind of work by doing something that's irresponsible.” - I15*
Forming interpersonal trust over time	*“I have a number of natural area sites [...] that I am with long-term. I've been with each of those cities for 10 years. I really get to know not only the habitat and the natural area itself that I'm taking care of, but also the neighbors around it. People that use that natural area, whether they walk their dogs or their property abuts [it]. Do they have concerns about fire and ladder fuels or they have concerns about weeds? I kind of hear all of it. I end up forming pretty good relationships with a lot of the landowners around the public natural areas. Or somebody will tell their friend about me, and I'll get a phone call from a friend of a friend who has a natural area or wildlife question. And so, I feel like it's during the course of my job, because I'm on these sites, I form pretty strong relationships with a lot of the community members that are around them. And so, they feel very comfortable and free to come and ask me about what's going on, what are the goals of the restoration, how can they be involved? And those are really easy places for me to hear about any concerns about beavers specifically or to let folks know, ‘Hey, we recently saw beaver chew in the area. This is what it looks like. This is what it means for you, and this is what is really cool about beavers. However, if it reaches a point where they're taking down [trees] or causing unsustainable damage, then give us a call and we'll see what we can do about it.’” - I22*

### Insight 2: Practitioners Built Trust Throughout the Adaptive Management Cycle

**Fig. 1:**
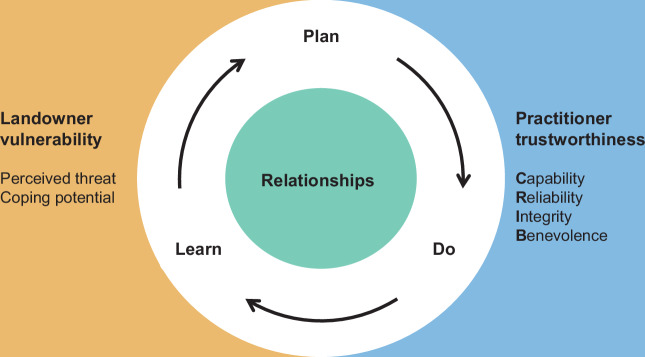
Conceptual diagram of trust-building tactics across the adaptive management cycle. Tactics address landowner vulnerability (orange), practitioner trustworthiness (blue), and the relationship (green) between landowner and practitioner

We identified 60 tactics for building trust and relationships with landowners based upon interviews with practitioners. These tactics aligned with the Shared Foundations Model by demonstrating the four dimensions of **trustworthiness** (capability, reliability, integrity, and benevolence), by reducing landowner **vulnerability** through managing perceived threats and maintaining or increasing coping potential, and by focusing on **relationships** more broadly. Trust-building tactics were used across the plan-do-learn cycle of adaptive management, with different dimensions emphasized at different stages (Fig. 1, Online Resource 2). Notably, tactics often addressed multiple trust dimensions simultaneously. For simplicity and clarity, we focus on what we consider to be the primary dimension of trust being targeted by each tactic, but acknowledge some redundancy between tactics that demonstrate trustworthiness, reduce vulnerability, and cultivate relationships.

#### Demonstrating the Trustworthiness C.R.I.B

##### Capability

**Fig. 2:**
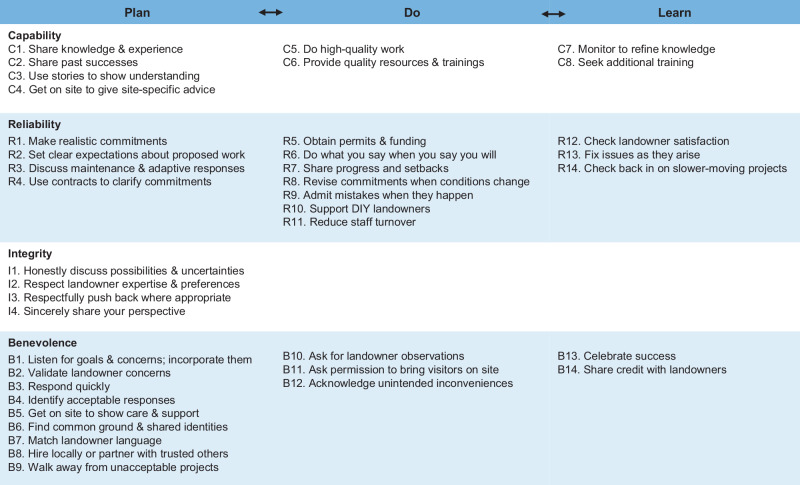
Tactics for demonstrating practitioner trustworthiness. Tactics are organized according to which aspect of the trustworthiness C.R.I.B. they primarily address and which stage of the adaptive management cycle they were described in

During **planning**, practitioners shared their knowledge about beavers and possible solutions through phone consultations and site visits (Fig. 2). Getting on site allowed practitioners to gain an in-depth understanding of the situation and make more refined predictions of the impacts of beaver activity and various human actions. Talking through scenarios with landowners while onsite showed practitioners’ nuanced understanding of beavers and riparian systems as well as landowners’ management realities. Practitioners also shared their successful experiences with nonlethal mitigation and restoration through stories, pictures, videos, and tours of other projects. During **implementation**, practitioners showed capability by completing high-quality work and providing quality resources and trainings. In the **learning** phase, monitoring allowed practitioners to refine their knowledge of how systems respond to interventions and which techniques work best in various contexts. This experience-based learning allowed practitioners to continually improve their beaver-related restoration capability. Outside of interactions with landowners, practitioners also sought additional training in nonlethal beaver conflict mitigation techniques, including through the Beaver Institute.

##### Reliability

During **planning**, practitioners managed landowner expectations and made clear commitments they could keep (Fig. 2). They avoided overpromising about the effectiveness of nonlethal mitigation, noting that solutions often required ongoing maintenance, could require adaptation, and may create new problems. For restoration work, they emphasized an “if you build it, [beavers] will come” (I09) approach that created suitable habitat and allowed dispersing beavers to eventually colonize sites rather than promising immediate, positive results. Practitioners positioned themselves as partners that landowners could count on who would be there for landowners if or when surprises came up. When substantial resources (time or money) were involved, formal and informal contracts were often used to clarify commitments from all parties. During **implementation**, demonstrating reliability meant following through. When supporting Do-It-Yourself (DIY) landowners, practitioners taught landowners to recognize signs of beaver activity, think like a beaver, and the principles behind effective mitigation devices. When doing work themselves, practitioners stayed in communication with landowners, provided updates as work progressed, and involved landowners when the situation changed and adaptations were needed. These conversations updated commitments and avoided surprising landowners. When things went wrong, such as when beavers built a new dam, exclusion devices did not work, or stream flow changed in problematic ways (e.g., flooding), practitioners maintained reliability by admitting their mistakes, taking responsibility, and working to correct them. Practitioners worked to reduce staff turnover so landowners would have consistent contacts throughout long-term projects. For slower moving projects, implementation involved checking back in. In the **learning** phase, practitioners ensured promises were kept by following up after implementation finished and fixing issues as they arose.

##### Integrity

Emphasized primarily in the **planning** stage, practitioners showed integrity through honest, forthright conversations that walked landowners through various possibilities of action or inaction (Fig. 2). Sometimes, they pushed back on landowner fears that beavers would cause catastrophic damage. At other times, practitioners discussed where beavers might appear and which changes may take place, checking in about whether landowners were tolerant of these potential changes. The beaver activity scenario discussion was sometimes described as gauging landowner tolerance for beavers. These conversations included discussions of uncertainty, with practitioners mentioning they expected surprises, but their adaptive approach meant they would monitor for and respond to changes. Practitioners also demonstrated integrity when they sincerely shared what they thought, even when they knew this went against the landowners’ perspectives or what they might want to hear (e.g., that landowners were making unreasonable demands, had unrealistic beliefs about the consequences of beaver activity). They did so while also respecting landowners’ values, interests, and experience, which was especially important when landowner and practitioner perspectives diverged. Thus, being honest about what might happen and sincerely sharing the practitioner’s perspective went together to show that practitioners meant what they said and could be counted on to tell the truth, even when it might go against their restoration goals. Although integrity was discussed less often related to **implementation** and **learning** phases, practitioners were honest and forthright as they employed reliability-targeted tactics, such as admitting mistakes and discussing adaptation needs (see above).

##### Benevolence

During **planning**, practitioners dedicated themselves to understanding landowners’ perspectives through empathic listening, validating concerns, and uncovering landowner priorities (Fig. 2). They found common ground and emphasized shared identities. This included hiring local staff, partnering with trusted organizations, and matching landowner language. It also meant finding mutually beneficial solutions instead of persuading landowners into something that went against their interests. At times, this meant being willing to walk away when interests diverged. In addition to showcasing practitioners’ capability, site visits were powerful ways for practitioners to demonstrate their benevolence, namely that they cared enough to respond quickly and take time for meaningful interactions with landowners. Although it is listed last in the trustworthiness CRIB, demonstrating benevolence was perhaps the most heavily emphasized area for practitioners’ early trust-building efforts even more so than capability, reliability or integrity.

During **implementation**, practitioners asked permission before bringing other people onto properties, took responsibility for consequences, and acknowledged unintentional impacts. For example, one practitioner gave herbicide as a gift after traversing the property many times, acknowledging they could have spread weeds in the process. In the **learning** phase, practitioners shared credit with landowners, celebrated successes and featured them in presentations and newsletters. This was intended to help landowners feel positively about their contributions while also recruiting new landowners. Additionally, by checking in on landowners’ satisfaction, practitioners showed they cared whether landowners’ goals were met and their concerns addressed.

#### Addressing Landowner Vulnerability

**Fig. 3:**
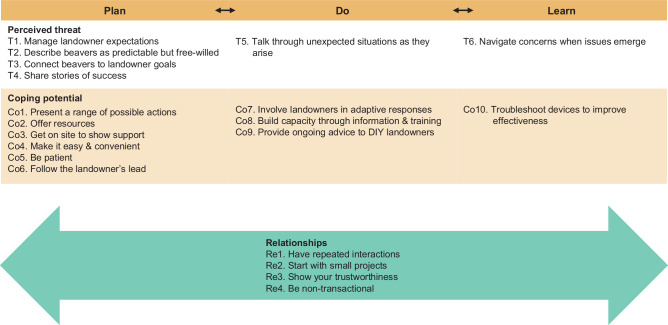
Tactics for addressing landowner vulnerability and building relationships across the adaptive management cycle. Vulnerability-focused tactics (orange) address landowners’ perceived vulnerability and coping potential. Relationships tactics (green) span the stages of adaptive management

Many of the trust-building tactics that demonstrated restoration practitioner trustworthiness also targeted components of landowner vulnerability: perceived threat and coping potential (Fig. 3, Online Resource 2).

##### Perceived threats

During **planning**, practitioners worked to distinguish between realistic expectations and unrealistic fears or hopes (Fig. 3). For landowners fearing beaver activity, practitioners reframed perceived damage as frequently tolerable, emphasized the predictability of beaver behavior, and noted alignment between the benefits of beavers and landowner goals. They made unfamiliar risks less threatening by sharing stories and visuals of successful past responses in similar situations. For landowners with overly optimistic hopes for beaver-based restoration, practitioners provided honest assessments about timelines, availability of quality habitat, uncertainty of riparian system responses, and described beavers as predictable but free-willed wild animals (i.e., non-controllable). During **implementation**, when surprising or unexpected things happened, practitioners returned to planning-stage tactics by talking through what was happening and strategizing acceptable and effective responses with landowners. Our interviewees believed that this ongoing dialog helped prevent new developments from escalating into major threats. Similarly, during the **learning** phase, if mitigation devices proved ineffective, beavers moved to new areas or created new issues, or restoration created intolerable changes, practitioners again engaged in planning-stage conversations to talk through potential solutions and navigate landowner concerns.

##### Coping potential

During **planning**, restoration practitioners focused on helping landowners see the options available and building landowners’ capacity to respond (Fig. 3). They presented themselves as available resources for funding, implementation, and expert advice when surprises arose or new needs emerged. Site visits helped communicate that landowners were not alone and that practitioners could respond should the situation become intolerable. Practitioners offered a range of possible actions and made approaches easy and convenient while being patient with landowner decision-making timelines. During **implementation**, practitioners increased landowner coping potential by providing information and training that improved landowners’ ability to address beaver issues themselves, including supporting DIY landowners with ongoing advice. Interviewees described how, in their experience, this approach helped landowners move from feeling overwhelmed to being able to respond adaptively, increasing their sense of control and efficacy in handling situations. During the **learning** phase, practitioners troubleshot mitigation and restoration devices that they and others built to help landowners be even better able to coexist with beavers.

An important part of increasing coping potential across stages was maintaining landowner autonomy by keeping control within landowners’ hands. Practitioners followed landowners’ lead, even if it meant projects moved slower or achieved less dramatic ecological changes. They described waiting for landowners to initiate next steps, generate ideas, and make decisions, even if it took months or years. Patience was essential, and practitioners emphasized not pushing too hard to avoid losing landowners altogether. They acknowledged that current land management practices were learned over time and culturally ingrained, and that sometimes landowners needed their attempts to fail before they’d be willing to consider alternative approaches. Generational shifts or new landowners could bring new opportunities; however, practitioners also acknowledged that such changes did not guarantee openness to new approaches. Another part of following the landowner’s lead was to match the intervention to landowners’ desired level of involvement. Some landowners wanted others to fix their problem, other landowners preferred to respond on their own (the DIY solution mentioned above), and still others fell somewhere in between. In these instances, practitioners focused on empowering landowners to pursue whichever approach they preferred.

#### Building Trust Through Relationships

Practitioners emphasized that trust was often built through relationships, which either served as the trust-building mechanism or enabling other trust-building tactics to be effective. Relationship building occurred across the adaptive management cycle, but it often extended beyond formal project timelines, sometimes beginning well before a project was envisioned and continuing long after its official completion. Thus, the relational tactics described here transcend the adaptive management cycle (Fig. 3, Online Resource 2).

Practitioners described repeated interactions with landowners that ranged from brief conversations to decades-long exchanges. Many of these relationships arose from living and working in small, rural communities where familiarity and shared experiences accumulated over time. Repeated interactions served several purposes. They allowed practitioners and landowners to build mutual familiarity, understand each other’s histories and interests, and identify shared values or identities that created common ground and a general liking for each other. Importantly, practitioners described getting to know landowners as people with their own stories, families, and interests, not just as project partners. This human-focused connection helped shift interactions from transactional exchanges furthering practitioner goals to opportunities to build genuine, reciprocal relationships grounded in care and respect.

A few practitioners also described starting with small projects. This allowed landowners to make low-risk commitments and observe how practitioners worked, which helped them assess practitioner trustworthiness. Meanwhile, practitioners used these early interactions to gauge landowner motivations and openness to future collaboration before fully committing to large-scale projects with unknown partners.

### Insight 3: Practitioners Navigated Tensions Surrounding Trust-building Tactics

Although they recognized trust-building as crucial for landowner engagement and outlined dozens of strategies they used to build trust, practitioners still described facing at least two enduring tensions when building trust with landowners: (1) individual trust-building tactics could compete with each other, and (2) different people responded to the same tactics in different ways.

First, practitioners described making difficult decisions about how best to communicate when two or more trust-building tactics were in conflict (Table 3). For example, showing one’s own expertise (capability) could conflict with respecting landowner knowledge and management practices (benevolence). Some interviewees noted that the more they emphasized their own knowledge, the more likely they felt they were to come across as disrespectful, not listening, and not valuing landowner knowledge and experience. Another example involved matching landowner language. Done well, interviewees felt that this could improve communication and imply shared values and identity (benevolence). Done poorly, the practitioner risked being seen as an insincere outsider (lack of integrity and benevolence). Similarly, demonstrating listening to and understanding of landowner concerns (benevolence) could conflict with sincerely sharing one’s perspective (integrity) and expertise (capability). Practitioners also debated whether to share their thoughts on controversial topics (integrity), which risked communicating a lack of shared values (benevolence).

**Table 3:** Tensions between trust-building tactics

Tension^a^	Quote
C1. Share knowledge & experience B3. Respect landowner expertise, preferences	*“It's all, 100%, about trust, not overstepping boundaries, not pointing out the fact that what [the landowner is] doing is making the landscape harder for [them] to do what [they’re] doing. It's really delicate topics […] people's livelihood in the ranching community relies on ecological health, and yet what they're doing isn't always the best thing for ecological health. It's like you're telling them their religion is wrong, and everything that they base their life on is backwards or something like that. That's what it would feel like to them. And so, to suggest different things can be really difficult.” I08*
B7. Match landowner language I3. Sincerely share your perspective	*“I just speak whatever language they find most accessible. I'll tell them exactly the same thing. And the thing with folks on the [political] right, especially, if I was going to paint with a broad brush is, you better not be two-faced. They better not get the impression that you're telling them one thing, and you tell somebody else another. And I don't do that, but what I do is I'll cater my vocabulary, and I'll cater my examples to something that I think folks will find more accessible.” I05*
B1. Listen for landowner goals and concerns I3. Sincerely share your perspective C1. Share knowledge & experience	*“I think every one of the calls I've gotten so far, except for maybe [one], starts with they want us to trap the beavers and move them. Most people don't want them killed, but they want them gone from their land, or they're trying to ask us if they're allowed to trap them. And so, I also try to make it clear that ‘yes, you are allowed to trap them. That's your right now under the law to do that. You're totally allowed to do that. And I don't think that'll solve your problem because beaver populations have increased. Your spot looks like a good spot for beavers and they'll come back.’ But I've found that enforcing their ability to trap them if they want to is really important, rather than just saying, ‘Here's all the reasons you shouldn't trap,’ starting with, ‘Hey, if you want to trap, you're totally allowed to. You can go for it. Here's why I think this other thing would be a better solution.’ And after the conversations, there's only been one landowner that still wanted to trap after that.” I04*
I3. Sincerely share your perspective B6. Find common ground, shared identities	*“We do talk about trapping sometimes. It's a weird divisive thing, even amongst beaver fanatics [...] it's a controversial thing, but we do talk about how the benefits of beavers increase by having sustained beaver occupation of land. If you're taking out beaver dams all the time, then the water quality benefits that we love aren't actually happening [...] At this point, we do not say that trapping is bad or great. I mean, we might think that or whatever. It's like, wetlands are supported by a lot of hunters and fishers, and beaver trapping is in that category. And so, I think that that's something that we are a little bit more careful about. I appreciate the issue, personally, but it's something where we will not tell people that trapping is bad, but also that there are maybe some places where trapping should not happen and it should be regulated and we believe that there can be ways to do this that are better for the environment.” I09*

^a^Tactic labels, e.g. C1, identified in figures 2 and 3.

Second, practitioners also described having to navigate the reality that the same tactic might build trust with one landowner and reduce it with another person or in a different situation. For example, several practitioners described being patient and letting landowners lead the way as a key tenet of their approach to promoting beaver coexistence. However, this meant that the landowner might not contact them for six to twelve months or more. At this point, the practitioner had to decide whether to contact the landowner again or continue waiting. Reaching out to the landowner could be interpreted by landowners in multiple ways:

1. Attempting to ensure the practitioner kept their commitment to reach out (reliability);
2. Caring about the landowner enough to think about them and see if their concerns remained (benevolence); or
3. Impatiently pushing the practitioner’s own agenda (lack of benevolence).

Thus, practitioners had to anticipate how a specific landowner might respond in a particular situation and then read verbal and nonverbal cues to decide when to adjust their approach. These examples of tensions highlight how trust-building while promoting beaver coexistence requires practitioners to be attuned to individual landowners and situational contexts. Practitioners’ stories demonstrated that they must constantly adapt their approach based on real-time assessment of how their actions are being interpreted.

## Discussion

Trust-building emerged as an essential feature of practitioner communication with private landowners when promoting beaver-related restoration and coexistence. Instead of being an addition, trust was how much of their restoration work got done. This finding reinforces past research showing trust and relationship building is essential for private lands conservation work (Auster et al. 2021; Lute et al. 2018; Metcalf et al. 2015).

Beaver-based restoration asks private landowners to voluntarily choose to embrace change, a choice that creates varying degrees of vulnerability for landowners (Metcalf et al. 2015). For some, this means reconsidering existing practices of lethally removing beavers even though current lethal responses are rooted in decades of managing beavers as pests and shaped by local expectations around what responsible land stewardship looks like (Jonker et al. 2009; Morzillo and Needham 2015). For others who want to attract beavers, the appeal of increasing water storage, restoring wetlands, or bringing nature back can hide the realities of living alongside a free-willed ecosystem engineer. In both situations, beaver activity introduces new hydrological dynamics, changes vegetation, and influences land use (Brazier et al. 2021). Such changes may challenge norms around how landscapes should look or function, especially in places where human infrastructure sits atop former wetlands or where historical beaver extirpation during the fur trade has influenced local understandings of which species “should” be present.

Voluntarily choosing change that challenges local norms carries risks. For example, news of a failed project can spread quickly in small, tight-knit rural communities and discourage community members from considering similar efforts on their land. When practitioners enter these settings, especially when they come from outside of a landowner’s community of place or professional background, they are asking landowners to take a risk, try something unfamiliar and uncertain, and trust a process that depends on adaptation rather than control. This context helps explain why trust-building is so important for coexistence and restoration work (Metcalf et al. 2015).

Environmental and natural resource management trust literature repeatedly shows that trust predicts desirable outcomes, with researchers often emphasizing that trust is important for conservation and environmental management and should be increased (Auster et al. 2021; Cvetkovich and Winter 2003; Liljeblad and Borrie 2006; Stern 2008; Stern and Coleman 2015; Toman et al. 2021). In comparison, the practitioners we interviewed told us, unprompted, that they already knew trust was important and described using a wide range of tactics to build it. Their experience suggests that future research, rather than continuing to examine whether trust is important, could focus on which trust-building tactics are effective in which situations, for which landowners, and why. To support this shift in research focus, our results point toward two overarching implications: (1) trust-building is a craft that can be mastered, and (2) trust repair is a part of that craft. Both of these insights highlight the need for research that can help practitioners refine their trust-building efforts in the complex and changing contexts in which beaver-related restoration and coexistence work takes place.

### Trust-building as a Craft

Practitioner trust-building efforts involve listening to landowners and responding appropriately (Auster et al. 2021; Erickson and Jones 2026). This requires situational awareness, interpersonal sensitivity, and real-time judgment. As such, we interpret trust-building not as a routinized set of steps but as a practice-based craft, similar to science communication (Harmon and Gross 2010) and science facilitation (Cravens et al. 2022), that is developed over time through experiences with diverse landowners, contexts, and outcomes.

Viewing trust-building as a craft suggests that these skills are likely to develop along a general progression, beginning with more intuitive tactics and becoming refined with experience. Novice trust-builders may realize that trust matters for successful restoration projects and focus on straightforward but essential tactics, such as communicating honestly (tactic I1) (Erickson and Biedenweg 2025; Mayer et al. 1995) as well as connecting beavers to landowner goals (tactic T3) (Charnley et al. 2020), and sharing practitioners’ knowledge about beavers and mitigation (tactic C1) (Erickson and Jones 2026). They may be most effective with landowners who share the practitioner’s values and worldviews (Cvetkovich and Winter 2003) but may have fewer strategies for responding when trust is violated. With additional experience, practitioners may expand their repertoire, becoming better at listening and empathizing with landowners (tactics B1, B2) (Auster et al. 2021; Erickson and Jones 2026), and anticipating ways projects may fail (tactics B4, B9). They may focus on managing expectations (tactics T1, T2) (Feltman 2021), discussing tradeoffs (tactic I1) (Charnley et al. 2020), making realistic commitments (tactic R1) (Briccetti et al. 2025; Feltman 2021), and providing ongoing support when surprises arise or mitigation devices fail (tactics Co10, R10, R13). Intermediate trust-builders may improve at tailoring their communication to their audience (tactic B7), respectfully correcting misconceptions (tactic I3), and maintaining trust after minor violations. More advanced practitioners may be able to anticipate misunderstanding earlier and adjust proactively to prevent predictable missteps. At this stage, practitioners may also be able to connect with audiences with very different priorities and worldviews (tactic B6), and they may be skilled at recovering from more severe trust violations. They may also be able to successfully build trust after initiating contact with a landowner, an action that can lead landowners to suspect practitioners hold ulterior motives.

Conceptualizing trust as a craft highlights several directions for future research. Researchers could examine how practitioners use specific tactics in real-world settings, including which tactics are used more or less often and how their use varies across practitioner roles, landowner audiences, and project contexts. Another line of research could evaluate the effectiveness of tactics with different audiences, such as skeptical landowners, new landowners, or those with varying management goals or histories. A third direction could study how practitioners’ trust-building skills develop over time, testing whether the hypothetical progression above matches real-world practice, whether development of trust-building mastery varies by sector (nonprofit, government, for-profit) or specialty (habitat restoration vs wildlife conflict mitigation), and whether capacity-building interventions can accelerate the development of trust-building expertise.

### Trust Repair as Part of the Craft

Practitioners described beaver-related restoration as often unfolding over long timeframes, from months to years or even decades. During this time, ecological and hydrological conditions are changing in response to interventions, and social conditions may change as well (e.g., new neighbors, changing levels of tolerance for beavers, new incentive programs, new staff at agencies and organizations). Moments are likely to arise where trust is strained or violated. Navigating trust with private landowners while promoting beaver-related restoration, then, likely includes building, maintaining, and repairing trust. This conceptualization runs counter to popular narratives about trust taking “years to build, seconds to break, and forever to repair” (Wilson 2025).

Trust can be strained during beaver-related restoration due to ecological surprises (e.g., beavers behaving unexpectedly), technical setbacks (e.g., devices failing), and communication challenges (unmet or differing expectations). Indeed, beavers, the very animals that are praised for bringing about ecological transformations, can also be the cause of trust violations. During interviews, practitioners described beavers and beaver-related restoration paradoxically as both predictable and unpredictable. Beavers’ needs for safety, shelter, food, and space allow experts to anticipate beavers’ likely behavior. At the same time, beavers, as creatures with their own motivations, can make choices that surprise practitioners (e.g., building a dam in an unexpected location, avoiding a site that appears to have enough food and quality habitat), and their activities can lead to unexpected outcomes (e.g., flooding, site abandonment). Similarly, practitioners described mitigation devices (e.g., exclusion fencing and pond levelers) as being effective and cost-saving but often requiring troubleshooting, customization, and maintenance. Such realities conflicted with potential landowner desires for a quick, cheap, and completely effective one-off solution.

Although practitioners did not use the term “trust repair,” they described tactics for anticipating and responding to surprises, disappointments, and unmet expectations. Their planning stage tactics emphasized managing expectations (tactic T1), explaining that adaptation and surprises were part of the process (tactic T2, R3), and understanding landowner goals (tactic T3, B1) and levels of tolerance (tactic B4). During implementation, practitioners described tactics such as acknowledging what happened (tactic B12), admitting mistakes (tactic R9), explaining causes (tactic T5), adjusting plans (tactic R8), teaching landowners to troubleshoot their devices (tactic R10), and showing up quickly when things went wrong (tactic R13). These tactics represent but a few of the trust repair tactics studied in other fields such as organizational behavior, psychology, and communication (Dirks et al. 2009; Gillespie et al. 2014; Gillespie and Dietz 2009; Hornsey et al. 2024; Kim et al. 2009; Lewicki and Brinsfield 2017; Reynolds and Lander 2024; Sharma et al. 2023; Tomlinson 2025).

Although trust repair is extensively developed in other fields, it remains underexplored in environmental management contexts. Cvitanovic et al. (2021) offer an important contribution by outlining a trust repair sequence described by practitioners at the interface of environmental science and policy. Our findings suggest a need for more research into trust repair in the context of restoration and coexistence, including which repair strategies practitioners use, how landowners interpret different repair strategies, and which strategies are more or less effective in different contexts. Bridging existing research on trust repair with restoration practice could help practitioners recognize when trust can or cannot be restored (Schweitzer et al. 2006) and equip them to draw upon decades of trust repair research. Given the extended timeframes and inherent uncertainties of beaver-related restoration, trust repair may be an important but overlooked component of the trust-building craft.

### Limitations

Our results should be considered in light of our study’s limitations. As a qualitative study with a small sample of practitioners from a single U.S. state, our results may not generalize and are not meant to be prescriptive. Because we did not set out to explore trust, we did not specifically ask practitioners about their efforts to build trust. Thus, our study participants may have additional tactics not identified here and may not view all tactics described in this manuscript in the light of trust-building (in other words, they might employ these tactics for other purposes besides or in addition to trust-building). Additionally, we rely on practitioner self-reported behavior and experiential knowledge learned in the field while working with landowners. We did not observe their communication or behavior with landowners directly, nor did we examine how landowners interpret practitioners’ attempts to build trust. Furthermore, we reported the trust-building tactics used by our interviewees as a collective whole but did not examine which tactics were used by which individuals, how often, and how well. Research into each and all of these areas could uncover additional insights, including a progression for increasing trust-building mastery. Such questions were beyond the scope of this study, but we encourage our peers to examine such questions, especially if they help uncover nuance around when various trust-building and trust repair tactics are most effective.

## Supplementary information

Online Resource 1

Online Resource 2

## Data Availability

The data underlying this article is confidential and cannot be shared publicly; however, select quotations from interviews are provided in Online Resource 2.

## References

[CR1] Arkle RS, Pilliod DS (2015) Persistence at distributional edges: Columbia spotted frog habitat in the arid Great Basin, USA. Ecol Evol 5(17):3704–3724. 10.1002/ece3.1627. 26380699 10.1002/ece3.1627 PMC4567874

[CR2] Auster RE, Barr SW, Brazier RE (2021) Improving engagement in managing reintroduction conflicts: Learning from beaver reintroduction. J Environ Plan Manag 64(10):1713–1734. 10.1080/09640568.2020.1837089.

[CR3] Baer MD, Colquitt JA (2018) Why do people trust? Moving toward a more comprehensive consideration of the antecedents of trust. In R. H. Searle, A.-M. I. Nienaber, & S. B. Sitkin (Eds.), *The Routledge companion to trust* (pp. 163–182). Routledge. 10.4324/9781315745572-12

[CR4] Beaver Management Work Group (2022) *Recommendations for beaver management on federal lands*. Prepared by Kearns & West for the Oregon Fish and Wildlife Commission.

[CR5] Bennett NJ, Dodge M, Akre TS, Canty SWJ, Chiaravalloti R, Dayer AA, Deichmann JL, Gill D, McField M, McNamara J, Murphy SE, Nowakowski AJ, Songer M (2022) Social science for conservation in working landscapes and seascapes. Front Conserv Sci 3: 954930. 10.3389/fcosc.2022.954930.

[CR6] Bernard HR (2006) *Research methods in anthropology: Qualitative and quantitative approaches*. AltaMira Press. 10.1007/978-3-662-49355-7_2

[CR7] Brazier RE, Puttock A, Graham HA, Auster RE, Davies KH, Brown CML (2021) Beaver: Nature’s ecosystem engineers. WIREs Water 8(1):e1494. 10.1002/wat2.1494. 33614026 10.1002/wat2.1494 PMC7883483

[CR8] Briccetti LH, Coleman KJ, Taylor LE (2025) “They’re the ones that sent me down a flooded road”: Trust, distrust, and individuals’ flood mitigation decisions. J Environ Planning Manag 1–23. 10.1080/09640568.2025.2524444

[CR9] Broman D, Wolfer B (2024) *2024-2026 furbearer regulations*. Oregon Fish and Wildlife Commission meeting. https://www.dfw.state.or.us/agency/commission/minutes/24/06_Jun/F/Ex%20F-Presentation-Furbearer%20Regulations.pdf

[CR10] Burgher JAS, Hallza J, Lambert MR, Piovia-Scott J (2026) Beaver-related restoration and freshwater climate resilience across western North America. Restor Ecol 34(1):e70194. 10.1111/rec.70194.

[CR11] Cafferata F, Petro V, Taylor J, Woodward J (2023) *Wildlife in managed forests: The North American beaver*. Oregon Forest Resources Institute.

[CR12] Charnley S (2019) *If you build it, they will come: Ranching, riparian revegetation, and beaver colonization in Elko County, Nevada* (p. 39). Res. Pap. PNW-RP-614. Portland, OR: U.S. Department of Agriculture, Forest Service, Pacific Northwest Research Station.

[CR13] Charnley S, Gosnell H, Davee R, Abrams J (2020) Ranchers and beavers: Understanding the human dimensions of beaver-related stream restoration on western rangelands. Rangel Ecol Manag 73(5):712–723. 10.1016/j.rama.2020.04.008.

[CR14] Cravens AE, Jones MS, Ngai C, Zarestky J, Love HB (2022) Science facilitation: Navigating the intersection of intellectual and interpersonal expertise in scientific collaboration. Humanities Soc Sci Commun 9(1):256. 10.1057/s41599-022-01217-1.

[CR15] Cvetkovich GT, Winter PL (2003) Trust and social representations of the management of threatened and endangered species. Environ Behav 35(2):286–307. 10.1177/0013916502250139.

[CR16] Cvitanovic C, Shellock RJ, Mackay M, van Putten EI, Karcher DB, Dickey-Collas M, Ballesteros M (2021) Strategies for building and managing ‘trust’ to enable knowledge exchange at the interface of environmental science and policy. Environ Sci Policy 123(June):179–189. 10.1016/j.envsci.2021.05.020.

[CR17] Davenport MA, Leahy JE, Anderson DH, Jakes PJ (2007) Building trust in natural resource management within local communities: A case study of the Midewin National Tallgrass Prairie. Environ Manag 39(3):353–368. 10.1007/s00267-006-0016-1. 10.1007/s00267-006-0016-1 17253093

[CR18] Dietz G, Den Hartog DN (2006) Measuring trust inside organisations. Pers Rev 35(5):557–588. 10.1108/00483480610682299.

[CR19] Dirks KT, Lewicki RI, Zaheer A (2009) Repairing relationships within and between organizations: Building a conceptual foundation. Acad Manag Rev 34(1):68–84. 10.5465/AMR.2009.35713285.

[CR20] Dubois A, Gadde L-E (2002) Systematic combining: An abductive approach to case research. J Bus Res 55(7):553–560. 10.1016/S0148-2963(00)00195-8.

[CR21] Emborg J, Daniels SE, Walker GB (2020) A framework for exploring trust and distrust in natural resource management. *Frontiers in Communication*, *5*. 10.3389/fcomm.2020.00013

[CR22] Emerson RM, Fretz, RI, Shaw LL (2011) *Writing ethnographic fieldnotes* (2nd ed.). The University of Chicago Press.

[CR23] Enck JW, Bishop PG, Brown TL, Lamendola JE (1992) *Beaver-related attitudes, experiences, and knowledge of key stakeholders in Wildlife Management Unit 21*. HDRU Series No. 92-7. Ithaca, NY.

[CR24] Erickson BD, Biedenweg K (2025) The shared antecedents of trust and distrust. *Society & Natural Resources*, 1–28. 10.1080/08941920.2025.2509306

[CR25] Erickson BD, Jones MS (2026) Synthesizing beaver coexistence messaging with the capability, opportunity, and motivation behavior model. Conserv Biol, e70210. 10.1111/cobi.70210 10.1111/cobi.70210 41502085

[CR26] Fairfax E, Westbrook C (2024) The ecology and evolution of beavers: Ecosystem engineers that ameliorate climate change. Annu Rev Ecol Evol Syst 55(1):323–345. 10.1146/annurev-ecolsys-102722-122317.

[CR27] Fairfax E, Whittle A (2020) Smokey the beaver: Beaver-dammed riparian corridors stay green during wildfire throughout the western United States. Ecol Appl 30(8):e02225. 10.1002/eap.2225. 32881199 10.1002/eap.2225

[CR28] Feltman C (2021) *The thin book of trust: An essential primer for building trust at work*. Thin Book Publishing Co.

[CR29] Fiske ST, Cuddy AJC, Glick P (2007) Universal dimensions of social cognition: Warmth and competence. Trends Cogn Sci 11(2):77–83. 10.1016/j.tics.2006.11.005. 17188552 10.1016/j.tics.2006.11.005

[CR30] Floyd DL, Prentice-Dunn S, Rogers RW (2000) A meta-analysis of research on protection motivation theory. J Appl Soc Psychol 30(2):407–429. 10.1111/j.1559-1816.2000.tb02323.x.

[CR31] Fountain SM (2014) Ranchers’ friend and farmers’ foe: Reshaping nature with beaver reintroduction in California. Environ Hist 19(2):239–269. 10.1093/envhis/emu003.

[CR32] Gillespie N, Dietz G (2009) Trust repair after an organization-level failure. Acad Manag Rev 34(1):127–145.

[CR33] Gillespie N, Dietz G, Lockey S (2014) Organizational reintegration and trust repair after an integrity violation: A case study. Bus Ethics Q 24(3):371–410. 10.5840/beq2014437.

[CR34] Goldfarb B, Flores D (2018) *Eager: The surprising, secret life of beavers and why they matter*. Chelsea Green Publishing.

[CR35] Gottschalk Druschke C, Booth EG, Demuth B, Holtgren JM, Lave R, Lundberg ER, Myhal N, Sellers B, Widell S, Woelfle-Hazard CA (2024) Re-centering relations: The trouble with quick fix approaches to beaver-based restoration. Geoforum 156: 104121. 10.1016/j.geoforum.2024.104121.

[CR36] Guziejka M (2025) *Urban beavers at the crossroads: How ecological knowledge, sociodemographics, and spatial context shape public perception and coexistence preferences* [Master of Science in Geography, Portland State University]. 10.15760/etd.3933

[CR37] Halley DJ, Saveljev AP, Rosell F (2021) Population and distribution of beavers *Castor fiber* and *Castor canadensis* in Eurasia. Mammal Rev 51(1):1–24. 10.1111/mam.12216.

[CR38] Harmon JE, Gross AG (2010) *The craft of scientific communication* (1st ed.). University of Chicago Press. 10.7208/9780226316635

[CR39] Higuera PE, Abatzoglou JT (2021) Record-setting climate enabled the extraordinary 2020 fire season in the western United States. Glob Change Biol 27(1):1–2. 10.1111/gcb.15388. 10.1111/gcb.15388 33048429

[CR40] Hood GA, Manaloor V, Dzioba B (2018) Mitigating infrastructure loss from beaver flooding: A cost–benefit analysis. Hum Dimens Wildl 23(2):146–159. 10.1080/10871209.2017.1402223.

[CR41] Hornsey MJ, Chapman CM, La Macchia S, Loakes J (2024) Corporate apologies are effective because reform signals are weighted more heavily than culpability signals. J Bus Res 177: 114620. 10.1016/j.jbusres.2024.114620.

[CR42] Horry County (2025) *Beaver reduction program*. HCGovernment. https://www.horrycountysc.gov/beaver-reduction-program/

[CR43] Jonker SA, Organ JF, Muth RM, Zwick RR, Siemer WF (2009) Stakeholder norms toward beaver management in Massachusetts. J Wildl Manag 73(7):1158–1165. 10.2193/2004-160.

[CR44] Kearns & West (2022) *Recommendations for beaver management on federal lands*. Report from the Oregon Fish and Wildlife Commission Beaver Management Work Group to the Oregon Fish and Wildlife Commission. https://dfw.state.or.us/agency/commission/minutes/22/05_may/Beaver%20Management%20Work%20Group%20Recommendations%20-%20Final%204-29-22.pdf

[CR45] Kentnesse L (2023) *HB 3464 A staff measure summary*. Senate committee on natural resources, 82nd Oregon Legislative Assembly, 2023 Regular Session. https://olis.oregonlegislature.gov/liz/2023R1/Measures/Analysis/HB3464

[CR46] Kim PH, Dirks KT, Cooper CD (2009) The repair of trust: A dynamic bilateral perspective and multilevel conceptualization. Acad Manag Rev 34(3):401–422. 10.5465/AMR.2009.40631887.

[CR47] Kinas H, O’Shaughnessy K, McLeod A (2024) *Alberta beaver beneficial management practices*. Prepared for Working with Beavers. https://www.workingwithbeavers.ca/files/Beaver_BMPs_v2.pdf

[CR48] Koenigsberg S (2018) *The beaver believers* [Video recording]. Tensegrity Productions. https://www.thebeaverbelievers.com/

[CR49] Korsgaard MA (2018) Reciprocal trust: A self-reinforcing dynamic process. In R. H. Searle, A.-M. I. Nienaber, & S. B. Sitkin (Eds.), *The Routledge Companion to Trust* (pp. 14–28). Routledge. 10.4324/9781315745572-3

[CR50] Korsgaard MA, Brower HH, Lester SW (2015) It isn’t always mutual: A critical review of dyadic trust. J Manag 41(1):47–70. 10.1177/0149206314547521.

[CR51] Kremen C, Merenlender AM (2018) Landscapes that work for biodiversity and people. Science 362(6412):eaau6020. 10.1126/science.aau6020. 30337381 10.1126/science.aau6020

[CR52] Lawton JH, Jones CG (1995) Linking species and ecosystems: Organisms as ecosystem engineers. In C. G. Jones & J. H. Lawton (Eds.), *Linking species & ecosystems* (pp. 141–150). Springer US. 10.1007/978-1-4615-1773-3_14

[CR53] Lazarus RS (1985) The psychology of stress and coping. Issues Ment Health Nurs 7(1–4):399–418. 3854019 10.3109/01612848509009463

[CR54] Lewicki RJ, Brinsfield C (2017) Trust repair. Annu Rev Organ Psychol Organ Behav 4:287–313. 10.1146/annurev-orgpsych-032516-113147.

[CR55] Liljeblad A, Borrie WT (2006) Trust in wildland fire and fuel management decisions. Int J Wilderness 12(1):39–43.

[CR56] Lute ML, Gillespie CR, Martin DR, Fontaine JJ (2018) Landowner and practitioner perspectives on private land conservation programs. Soc Nat Resour 31(2):218–231. 10.1080/08941920.2017.1376139.

[CR57] Mayer RC, Davis JH, Schoorman FD (1995) An integrative model of organizational trust. Acad Manag Rev 20(3):709–734.

[CR58] McEvily B, Tortoriello M (2011) Measuring trust in organisational research: Review and recommendations. J Trust Res 1(1):23–63. 10.1080/21515581.2011.552424.

[CR59] McKinstry MC, Anderson SH (1999) Attitudes of private- and public-land managers in Wyoming, USA, toward beaver. Environ Manag 23(1):95–101. 10.1007/s002679900170. 10.1007/s002679900170 9817774

[CR60] Metcalf EC, Mohr JJ, Yung L, Metcalf P, Craig D (2015) The role of trust in restoration success: Public engagement and temporal and spatial scale in a complex social-ecological system. Restor Ecol 23(3):315–324. 10.1111/rec.12188.

[CR61] Morzillo AT, Needham MD (2015) Landowner incentives and normative tolerances for managing beaver impacts. Hum Dimens Wildl 20(6):514–530. 10.1080/10871209.2015.1083062.

[CR62] Nash CS, Grant GE, Charnley S, Dunham JB, Gosnell H, Hausner MB, Pilliod DS, Taylor JD (2021) Great expectations: Deconstructing the process pathways underlying beaver-related restoration. BioScience 71(3):249–267. 10.1093/biosci/biaa165.

[CR63] Nie M (2003) Drivers of natural resource-based political conflict. Policy Sci 36(3):307–341.

[CR64] O’Brien BC, Harris IB, Beckman TJ, Reed DA, Cook DA (2014) Standards for reporting qualitative research: A synthesis of recommendations. Acad Med 89(9):1245–1251. 10.1097/ACM.0000000000000388. 24979285 10.1097/ACM.0000000000000388

[CR65] ODFW (2016) *Oregon conservation strategy*. Oregon Department of Fish and Wildlife. https://www.oregonconservationstrategy.org/overview/

[CR66] ODFW (2021) *Preliminary beaver-related analyses from ODFW’s Aquatic Inventories Program (AQI) data*. Beaver Management Working Group. https://www.dfw.state.or.us/wildlife/working_group/docs/beaver_management_Dec_8/OFWC%20AQI%20Preliminary%20Analysis%20BMWG%2012-8-21.pdf

[CR67] ODFW (2023) *ODFW’s 3-year action plan for beaver-modified landscapes*. Oregon Department of Fish and Wildlife.

[CR68] ODFW (2024a) *Oregon furbearer trapping and hunting regulations: July 1, 2024 through June 30, 2026*. https://myodfw.com/sites/default/files/2025-10/Furbearer_Regulations.pdf

[CR69] ODFW (2024b) *2024-2025 and 2025-2026 Oregon furbearer information summary and regulation proposals*. Oregon Department of Fish and Wildlife. https://www.dfw.state.or.us/agency/commission/minutes/24/06_Jun/F/Exhibit%20F_Attachment%203_2024-2026%20Oregon%20Furbearer%20Regulation%20Proposals.pdf

[CR70] ODFW (2025) *Living with wildlife: American beaver*. Oregon Department of Fish and Wildlife. https://www.dfw.state.or.us/wildlife/living_with/docs/beaver.pdf

[CR71] ODFW (2026) *Furbearer trapping and hunting*. Oregon Department of Fish and Wildlife. https://myodfw.com/articles/furbearer-trapping-and-hunting

[CR72] ODFW (n.d.) *Fact sheet: Oregon’s state mammal*. Oregon Department of Fish and Wildlife. https://www.dfw.state.or.us/conservationstrategy/docs/Beaver_factsheet.pdf

[CR73] Oregon Legislative Assembly (2023) *House bill 3464*. https://apps.oregonlegislature.gov/liz/2023R1/Measures/Overview/HB3464

[CR74] Oregon Values and Beliefs Center (2023) *2023 typology study*. Oregon Values and Beliefs Center. https://oregonvbc.org/political-typology-study/

[CR75] Patton MQ (2002) Qualitative designs and data collection. In *Qualitative research and evaluation methods* (pp. 207–257). Sage Publications.

[CR76] Pilliod DS, Rohde AT, Charnley S, Davee RR, Dunham JB, Gosnell H, Grant GE, Hausner MB, Huntington JL, Nash C (2018) Survey of beaver-related restoration practices in rangeland streams of the western USA. Environ Manag 61(1):58–68. 10.1007/s00267-017-0957-6. 10.1007/s00267-017-0957-6 29167949

[CR77] Pollock MM, Lewallen GM, Woodruff K, Jordan CE, Castro JM (2023) *The beaver restoration guidebook: Working with beaver to restore streams, wetlands, and floodplains. Version 2.02*. United States Fish and Wildlife Service. https://www.fws.gov/sites/default/files/documents/The-Beaver-Restoration-Guidebook-v2.02_0.pdf

[CR78] Population Research Center (2025) *2024 certified population estimates*. College of Urban and Public Affairs, Portland State University. https://www.pdx.edu/population-research/population-estimate-reports

[CR79] PytlikZillig LM, Hamm JA, Shockley E, Herian MN, Neal TMS, Kimbrough CD, Tomkins AJ, Bornstein BH (2016) The dimensionality of trust-relevant constructs in four institutional domains: Results from confirmatory factor analyses. J Trust Res 6(2):111–150. 10.1080/21515581.2016.1151359.

[CR80] Reynolds N-S, Lander MW (2024) From building and preserving to eroding trust: A multi-level analysis. Group Organ Manag 49(4):1012–1044. 10.1177/10596011231190155.

[CR81] Rogers RW (1975) A protection motivation theory of fear appeals and attitude change. J Psychol 91(1):93–114. 10.1080/00223980.1975.9915803. 28136248 10.1080/00223980.1975.9915803

[CR82] Rogers RW (1983) Cognitive and physiological processes in fear appeals and attitude change: A revised theory of protection motivation. In: Cacioppo InJT, Petty RE (Eds.) Social psychology: A sourcebook. Guilford Press, 153–176.

[CR83] Rousseau DM, Sitkin SB, Burt RS, Camerer C (1998) Not so different after all: A cross-discipline view of trust. Acad Manag Rev 23(3):393–404. 10.5465/AMR.1998.926617.

[CR84] Saldaña J (2016) *The coding manual for qualitative researchers*. Sage Publications.

[CR85] SAMHSA (2019) *Communicating in a crisis: Risk communication guidelines for public officials*. SAMHSA Publication No. PEP19-01-01-005. Rockville, MD, Substance Abuse and Mental Health Services Administration.

[CR86] Schweitzer ME, Hershey JC, Bradlow ET (2006) Promises and lies: Restoring violated trust. Organ Behav Hum Decis Process 101(1):1–19. 10.1016/j.obhdp.2006.05.005.

[CR87] Sharma K, Schoorman FD, Ballinger GA (2023) How can it be made right again? A review of trust repair research. J Manag 49(1):363–399. 10.1177/01492063221089897.

[CR88] Siegrist M (2000) The influence of trust and perceptions of risks and benefits on the acceptance of gene technology. Risk Anal 20(2):195–204. 10.1111/0272-4332.202020. 10859780 10.1111/0272-4332.202020

[CR89] Sitkin SB, Roth NL (1993) Explaining the limited effectiveness of legalistic “remedies” for trust/distrust. Organ Sci 4(3):367–392.

[CR90] Smith JW, Leahy JE, Anderson DH, Davenport MA (2013) Community/agency trust and public involvement in resource planning. Soc Nat Resour 26(4):452–471. 10.1080/08941920.2012.678465.

[CR91] Stern MJ (2008) The power of trust: Toward a theory of local opposition to neighboring protected areas. Soc Nat Resour 21(10):859–875. 10.1080/08941920801973763.

[CR92] Stern MJ, Coleman KJ (2015) The multidimensionality of trust: Applications in collaborative natural resource management. Soc Nat Resour 28(2):117–132. 10.1080/08941920.2014.945062.

[CR93] Tape KD, Clark JA, Jones BM, Kantner S, Gaglioti BV, Grosse G, Nitze I (2022) Expanding beaver pond distribution in Arctic Alaska, 1949 to 2019. Sci Rep 12(1):7123. 10.1038/s41598-022-09330-6. 35504957 10.1038/s41598-022-09330-6 PMC9065087

[CR94] Taylor J (2021) *Beaver 101*. ODFW Beaver Management Work Group. https://www.dfw.state.or.us/wildlife/working_group/beaver_management_211013.asp

[CR95] Thompson J (2022) A guide to abductive thematic analysis. *The Qualitative Report*. 10.46743/2160-3715/2022.5340

[CR96] Toman EL, Curtis AL, Shindler B (2021) What’s trust got to do with it? Lessons from cross-sectoral research on natural resource management in Australia and the U.S. Front Commun 5(January):527945. 10.3389/fcomm.2020.527945.

[CR97] Tomlinson EC (2025) Trust repair in politically polarized workplaces. Hum Resour Manag Rev 35(2):101071. 10.1016/j.hrmr.2024.101071.

[CR98] Tversky A, Kahneman D (1974) Judgment under uncertainty: Heuristics and biases. Science 185(4157):1124–1131. 17835457 10.1126/science.185.4157.1124

[CR99] Vaske JJ, Absher JD, Bright AD (2007) Salient value similarity, social trust, and attitudes toward wildland fire management strategies. Hum Ecol Rev 14(2):223–232.

[CR100] Weathersby J, Julian JP (2025) A broader view of conservation: Mapping nature and culture of working lands in the Texas Hill Country. Land 14(5):991. 10.3390/land14050991.

[CR101] Weiskopf SR, Rubenstein MA, Crozier LG, Gaichas S, Griffis R, Halofsky JE, Hyde KJW, Morelli TL, Morisette JT, Muñoz RC, Pershing AJ, Peterson DL, Poudel R, Staudinger MD, Sutton-Grier AE, Thompson L, Vose J, Weltzin JF, Whyte KP (2020) Climate change effects on biodiversity, ecosystems, ecosystem services, and natural resource management in the United States. Sci Total Environ 733: 137782. 10.1016/j.scitotenv.2020.137782. 32209235 10.1016/j.scitotenv.2020.137782

[CR102] Welden EA (2023) Conceptualising multispecies collaboration: Work, animal labour, and Nature-based Solutions. Trans Inst Br Geogr 48(3):541–555. 10.1111/tran.12593.

[CR103] Wilson J (2025) *21 trust quotes that hit deep and speak the truth*. Quotes Tribe. https://quotestribe.com/trust-quotes/

[CR104] Zhuang Y, Fu R, Lisonbee J, Sheffield AM, Parker BA, Deheza G (2024) Anthropogenic warming has ushered in an era of temperature-dominated droughts in the western United States. Sci Adv 10(45):eadn9389. 10.1126/sciadv.adn9389. 39504363 10.1126/sciadv.adn9389 PMC11540010

