# Supplementary material for: Trust-building as a Keystone Activity in Beaver-related Restoration Practice
Source: Environ Manage. 2026 Feb 20;76(4):110. doi: 10.1007/s00267-026-02400-9 (PMC12923489; doi:10.1007/s00267-026-02400-9)
Supplement: Supplementary file 1 — Online Resource 1 [file 267_2026_2400_MOESM1_ESM.docx]

Online Resource 1

**Trust-building as a keystone activity in beaver-related restoration practice**

Brian D. Erickson^1^ & Megan S. Jones^2^

1. Department of Fisheries, Wildlife, and Conservation Sciences, Oregon State University, Corvallis, OR, USA. ORCID: 0000-0001-6264-1821
2. U.S. Geological Survey, Oregon Cooperative Fish and Wildlife Research Unit, Oregon State University, Corvallis, OR, USA. ORCID: 0000-0002-4284-3650

**Corresponding Author:** Brian D. Erickson, [brian.erickson@oregonstate.edu](mailto:brian.erickson@oregonstate.edu)

*Environmental Management*. DOI: 10.1007/s00267-026-02400-9

The interview guide (below) was approved by the Oregon State University Institutional Review Board (HE-2023-550). Informed consent was obtained from all individual participants included in the study.

# Semi-structured interview guide

1. To start, will you briefly describe your background and connections to helping private landowners live with beavers?

Let’s shift to talking about how you communicate with landowners about beaver.

## Beaver damage

One of the common issues for private landowners is damage from beavers. This includes damage from chewing as well as flooding.

1. Do you ever talk with private landowners about damage from beaver? (If no, skip)
2. Tell me about how you communicate with private landowners **BEFORE** any damage occurs.
3. Tell me about how you communicate with private landowners **WHEN** they discover the damage.
4. How, if at all, do you communicate differently depending on the **type** of damage or what gets damaged?
   1. For example, girdling, felling, blocked culvert, road flooding.
   2. Flooding from a dam versus a blocked culvert.
   3. For example, crops, timber, fencing, or a meaningful tree is chewed.
   4. For example, crops, timber, pasture, a road, or a building is flooded.
5. What different kinds of private landowners do you communicate with about damage from beaver?
6. Tell me about how you communicate with private landowners **AFTER** they respond to the damage.
7. Is there anything you think you should be doing, or wish you were doing, to communicate with landowners about beaver damage that you aren’t doing now?

## Habitat restoration

Another common focus is to involve private landowners in beaver habitat restoration on private land.

1. Do you ever talk with private landowners about participating in beaver habitat restoration on their property? (If no, skip)
2. Tell me about how you communicate with private landowners **BEFORE** a habitat restoration project begins.
   1. Do you tend to initiate contact with the landowner, or do they contact you about restoring habitat for beaver?
   2. Participation is a vague term. When you talk with landowners about “participating” in habitat restoration, what kinds of things are people being asked to do?
3. What kinds of private landowners do you communicate with about habitat restoration?
4. Tell me about how you communicate with private landowners **DURING** a habitat restoration project.
5. Tell me about how you communicate with private landowners **AFTER** a habitat restoration project finishes.
6. Have you ever handled conflicts that have arisen because of beaver habitat restoration efforts?
   1. Tell me about your communication about conflict **before** the restoration project began?
   2. Tell me about your communication about conflict **during** the restoration project?
   3. Tell me about your communication about conflict **after** the restoration project ended?
   4. How did the conflict affect the restoration efforts, if at all?
7. Is there anything you think you should be doing, or wish you were doing, to communicate with landowners about beaver habitat restoration that you aren’t doing now?
8. Do you ever communicate with private landowners about artificial beaver dams as a way to attract beavers? Tell me about that. [Skip if just focused on mimicking beaver.]

Now we’re shifting to a few final questions as we wrap up.

1. Are there any other ways that you communicate with private landowners about living with beaver that we have not discussed? Tell me about those.
2. Is there anything you tried in the past, communication-wise, that you’ve stopped doing?
3. You’ve talked about communication around beaver damage and restoration. How do you know if your communication is effective or not? [How do you define effective communication?]
4. What do you wish was happening related to beavers in Oregon that’s not?
5. Do you have any examples of your communication with landowners that you’d be willing to share?
6. Before we wrap up, is there anything I forgot to ask you that I should have?

[stop recording]

1. Do you have any questions for me?

## Referrals

1. Now that we’ve finished the interview, and you have a better sense of what I am asking, I am curious who else you think I should talk to for this study?
   1. I’m looking for folks who take similar as well as very different approaches to communicating about beaver coexistence.
   2. Do you happen to know the best way to reach them (phone, email, etc.)?
   3. Is it okay if I tell them that you suggested I reach out?
2. Is it okay if I contact you in the future about participating in dialogues, workshops, and other studies related to beaver coexistence in Oregon?
